# Nephrocalcinosis in farmed salmonids: diagnostic challenges associated with low performance and sporadic mortality

**DOI:** 10.3389/fvets.2023.1121296

**Published:** 2023-04-20

**Authors:** Hana Minarova, Miroslava Palikova, Radovan Kopp, Ondrej Maly, Jan Mares, Ivana Mikulikova, Ivana Papezikova, Vladimir Piacek, Lubomir Pojezdal, Jiri Pikula

**Affiliations:** ^1^Department of Ecology and Diseases of Zoo Animals, Game, Fish and Bees, Faculty of Veterinary Hygiene and Ecology, University of Veterinary Sciences Brno, Brno, Czechia; ^2^Department of Infectious Diseases and Preventive Medicine, Veterinary Research Institute Brno, Brno, Czechia; ^3^Department of Zoology, Fisheries, Hydrobiology and Apiculture, Faculty of AgriSciences, Mendel University in Brno, Brno, Czechia

**Keywords:** aquaculture, rainbow trout, water quality, carbon dioxide, fish health, blood acid-base balance, acid-base

## Abstract

Disease conditions that involve multiple predisposing or contributing factors, or manifest as low performance and/or low-level mortality, can pose a diagnostic challenge that requires an interdisciplinary approach. Reaching a diagnosis may also be limited by a lack of available clinical profile parameter reference ranges to discriminate healthy fish from those affected by specific disease conditions. Here, we describe our experience investigating poorly performing rainbow trout (*Oncorhynchus mykiss*) in an intensive recirculation aquaculture, where reaching a final diagnosis of nephrocalcinosis was not as straightforward as one would wish. To list the issues making the diagnosis difficult, it was necessary to consider the creeping onset of the problem. Further diagnostic steps needed to ensure success included obtaining comparative data for fish blood profiles and water quality from both test and control aquacultural systems, excluding infections with salmonid pathogenic agents and evaluating necropsy findings. Major events in the pathophysiology of nephrocalcinosis could be reconstructed as follows: aquatic environment hyperoxia and hypercapnia → blood hypercapnia → blood acid-base perturbation (respiratory acidosis) → metabolic compensation (blood bicarbonate elevation and kidney phosphate excretion) → a rise in blood pH → calcium phosphate precipitation and deposition in tissues. This case highlights the need to consider the interplay between water quality and fish health when diagnosing fish diseases and reaching causal diagnoses.

## 1. Introduction

As the role of world fisheries and aquaculture in providing food, nutrition and employment in a sustainable manner increases, there is an increasing need to address the issues of intensification, environmental challenges, biosecurity and disease (1). Just as fish health closely reflects the aquatic environment (2), biosecurity and water quality issues go hand in hand with fish stocking density (3).

In recent years, aquacultural production has gradually come to equal, or even surpass, that of capture fisheries, in part due to the development and implementation of recirculating aquaculture systems (RAS) (4, 5). While it is possible to control water quality parameters that limit the effectivity of RAS, such as dissolved oxygen (O_2_) and nitrogen (N) levels or pH, increased levels of carbon dioxide (CO_2_) may still be an issue (5), with levels considered safe for salmonids [i.e., < 10–15 mg/L; (6, 7)] occasionally exceeded. RAS CO_2_ levels are primarily a function of fish respiration, stocking density and the level of O_2_ supplementation, used to promote fish growth by increasing their metabolic rate (5, 8). Use of groundwater may also contribute to higher CO_2_ exposure (9).

In fish, aquatic CO_2_ exerts adverse effects in a concentration- and exposure duration-dependent manner (10–14). The anesthetic effects of CO_2_ (associated with a reduction in blood pH that induces a decrease in oxygen transported to respiring tissues, including the central nervous system) can be used as an overdose to achieve fish euthanasia (15, 16), and has been used on a larger scale to depopulate closed aquatic habitats or to remove invasive and/or non-native fish species (17). Longer-term exposure to elevated CO_2_ levels results in behavioral disturbances (18), stress, lower feed intake and growth retardation (11–20), and can eventually cause pathological lesions of nephrocalcinosis due to mineral deposits in the kidney (6, 8, 11, 14, 21–27).

While the rainbow trout (*Oncorhynchus mykiss*) industry represents an important share of aquacultural production (28), the species is known for its poor tolerance to elevated CO_2_ levels (14, 17, 25). However, fish populations affected by nephrocalcinosis are often characterized by low mortality rates (14, 21, 22, 25). Instead, the condition manifests itself through low performance and subsequent economic losses in fish production (11–13); thus, this pathophysiological condition poses a diagnostic challenge as its initial development at the aquaculture facility may go unrecognized. While analysis of blood parameters may be a suitable diagnostic and health monitoring tool in aquaculture (22, 29–31), the approach has limitations associated with availability of blood profile parameter reference ranges for specific conditions and/or discrimination data from healthy control fish (22, 32).

Here, we describe our experience in reaching a diagnosis of nephrocalcinosis that occurred as a spontaneous event in farmed rainbow trout. As the acid-base imbalance is believed to be a central pathophysiological mechanism in nephrocalcinosis progression, we report on blood profile parameters sampled from both affected and healthy fish, gross and microscopic findings in fish, and water quality parameters.

## 2. Materials and methods

### 2.1. Fish health study

Our fish disease diagnostic laboratory (University of Veterinary Sciences Brno, Czech Republic) was contacted to provide advice with poorly performing salmonids in an intensive aquaculture. Available records mentioned sporadic cases of mortality at a farm using RAS tanks to produce rainbow trout. The fish population at the time of investigation was characterized by 207.39 ± 79.56 g body mass and 24.41 ± 2.86 cm body length. The RAS tanks were supplied with groundwater treated with ozone and the fish were fed T-EXTRA RANGE extruded pellets for growing trout, as recommended by the producer (Le Gouessant Aquaculture, Lamballe-Armor, France).

During an on-site visit, we evaluated general fish keeping and stocking conditions, performed a clinical examination, took fish blood-samples and, after euthanizing a sample of fish, collected specimens for gross and microscopic pathology examinations, as well as screening for viruses and bacterial and parasitic pathogens. Water samples for quality assessment were also obtained. At a later date, after suspecting non-infectious etiology, we collected comparative samples from a healthy neighboring aquacultural site (MENDELU; healthy fish measurements 162.94 ± 7.56 g and 17.52 ± 0.75 cm) supplied by a different water source.

### 2.2. Blood sampling and measurement

A total of 21 clinically diseased and eight healthy fish were sampled by puncturing caudalis vessels with an 18 G needle and drawing blood into a 3 mL heparinized polypropylene syringe. Within 30 s of blood collection, an i-STAT portable clinical analyzer with an EC8+ cartridge (Abaxis, USA) was used to obtain on-site measurements of the following blood profile parameters: sodium (Na, mmol/L), potassium (K, mmol/L), chloride (Cl^−^, mmol/L), total dissolved CO_2_ (tCO_2_, mmol/L), glucose (mmol/L), pH, partial pressure of CO_2_ (pCO_2_, kPa), bicarbonate (HCO_3_, mmol/L) and base excess (BE, mmol/L). Following transportation to the laboratory, the remaining part of each blood sample was used to determine red and white blood cell counts (T/L and G/L), hemoglobin (g/L), hematocrit (L/L), mean corpuscular volume (fL), and mean corpuscular hemoglobin (pg) and mean corpuscular hemoglobin concentration (g/L). In addition, following centrifugation to obtain plasma, calcium (Ca, mmol/L), phosphorus (P, mmol/L), triglycerides (mmol/L), cholesterol (mmol/L), and total protein (g/L) were measured spectrophotometrically using a Konelab 20i biochemical analyzer and commercial test kits (Biovendor, Czech Republic), as described elsewhere (33).

### 2.3. Pathology examination

Necropsy examination was performed shortly after fish euthanasia (executed through stunning by a blow to the back of the head and killing by transection of the spine) in accordance with Czech legislation for the protection of animals against cruelty (Law No. 246/1992) and with EU legislation (Directive 2010/63/EU revising Directive 86/609/EEC on the protection of animals used for scientific purposes), as approved by the Ethical Committee of the University of Veterinary Sciences Brno, Czech Republic (REFNO: ES_11-2022_Necas). Written informed consent for participation of their animals in this study was obtained from the managers of the aquacultural facility.

In the laboratory, the skin, gills, and body cavity organs of each fish were visually inspected for gross lesions. Samples of gill, skin and caudal kidney tissues were fixed in 10% buffered formalin for histopathological analysis. The tissues were embedded in paraffin wax and processed, with sections (5 μm) stained by hematoxylin-eosin (Sigma-Aldrich, St Louis, MO, USA) and von Kossa (Abcam, Cambridge, UK) for visualization of calcium deposits. The histopathological diagnosis of nephrocalcinosis was based on findings defined by Klykken et al. (34).

### 2.4. Screening for viruses and bacterial and parasitic pathogens

Pooled samples of spleen, heart and cranial kidney were tested for salmonid viral pathogens, including Novirhabdovirus piscine (viral haemorrhagic septicaemia virus, VHSV), Novirhabdovirus salmonid (infectious haematopoietic necrosis virus, IHNV), infectious pancreatic necrosis virus (IPNV), salmonid alphavirus-2 (SAV-2), and piscine orthoreovirus-3 (PRV-3) using conventional and real-time PCR, as described in Pojezdal et al. (35).

Microbiological examination was performed as described in Palikova et al. (36, 37). Briefly, bacteriology swabs were obtained from gills and the spleen during necropsy. These were then inoculated directly onto blood agar (Oxoid, UK) and Tryptone Yeast Extract Agar for improved detection of Flavobacterium spp. (Sigma-Aldrich, USA). After inoculation, agar plates were incubated at 18 °C for 2–5 days and evaluated for bacterial colony growth on a regular basis. Tissue cytology imprints were examined using Diff-Quik and Gram Stains (Abcam, UK).

All fish were also inspected for presence of parasites visible to the naked eye. Wet mounts scraped from the gills and body surface, and squash tissue preparations of gills and internal organs, were also inspected for parasites using light microscopy and appropriate magnification. Kidney samples were tested for *Tetracapsuloides bryosalmonae*, the causative agent of proliferative kidney disease in salmonid fish, using PCR methods as described elsewhere (38).

### 2.5. Water quality measurement

Aquatic quality parameters (water saturation by oxygen, pH and temperature) were measured *in situ* at the time of rainbow trout capture using an HQ40D portable multi-meter (Hach, Loveland, Colorado, United States). Carbon dioxide concentration was calculated from values of water temperature, acid neutralizing capacity and pH according to Zalud et al. (39) and other chemical parameters were determined using standard methods (40). A total of ten measurements were obtained at both the poorly performing salmonid aquaculture and the control aquaculture (MENDELU).

### 2.6. Data analysis

Once measured, the blood parameters were assigned to groups of diseased and healthy fish and compared using the Kolmogorov-Smirnov and Shapiro-Wilks tests, one-way analysis of variance (ANOVA) and the non-parametric Kruskal Wallis, Tukey's multiple comparison and Mann-Whitney U tests. Significance levels were accepted as either *p* < 0.05 or *p* < 0.01. Principal component analysis (PCA) was also used to discriminate between diseased and healthy fish, based on the blood parameters measured. All data were analyzed using Statistica for Windows^®^ 14.0 (StatSoft Inc., USA). The original blood parameter data measured in this study are available in Supplementary Table 1.

## 3. Results

While dissolved O_2_ and CO_2_ levels were much higher at the aquacultural facility with poor performance and mortality, pH, nitrate (NO_3_), and chloride (Cl^−^) were lower. Furthermore, there were elevated levels of nitrogen (N) compounds in the form of ammonium (NH_4_) and nitrite (NO_2_) at the affected site (Table 1).

**Table 1:** Water quality parameters comparing conditions at a poorly performing salmonid aquacultural facility and those at a control facility (MENDELU), based on ten measurements at each farm.

**Parameter**	**Poor performance facility**	**Control facility (MENDELU)**
Temperature (°C)	14.40	15.50
O_2_ (%)	**129.30**	102.40
pH	**6.97**	7.85
CO_2_ (mg/L)	**10.68**	1.05
ANC (mmol/L)	0.85	0.65
N-NH_4_ (mg/L)	**0.09**	> LOD
N-NO_2_ (mg/L)	**0.17**	0.06
N-NO_3_ (mg/L)	**53.30**	98.00
P-PO_4_ (mg/L)	1.83	1.91
Cl^−^ (mg/L)	**75.52**	133.80
Ca^2+^ (mg/L)	77.15	61.13

ANC, acid-neutralizing capacity. LOD for N-NH_4_ is 0.01 mg/L. Numbers in bold are significantly different at *p* < 0.05.

Pooled samples of spleen, heart and cranial kidney tested negative for salmonid viral pathogens in all fish. Likewise, the fish had no obligate bacterial pathogens and only low-intensity parasite infection, stained kidney cytology imprints proved negative for *Renibacterium salmoninarum*, and the samples were also negative for *Tetracapsuloides bryosalmonae*.

**Figure 1:**
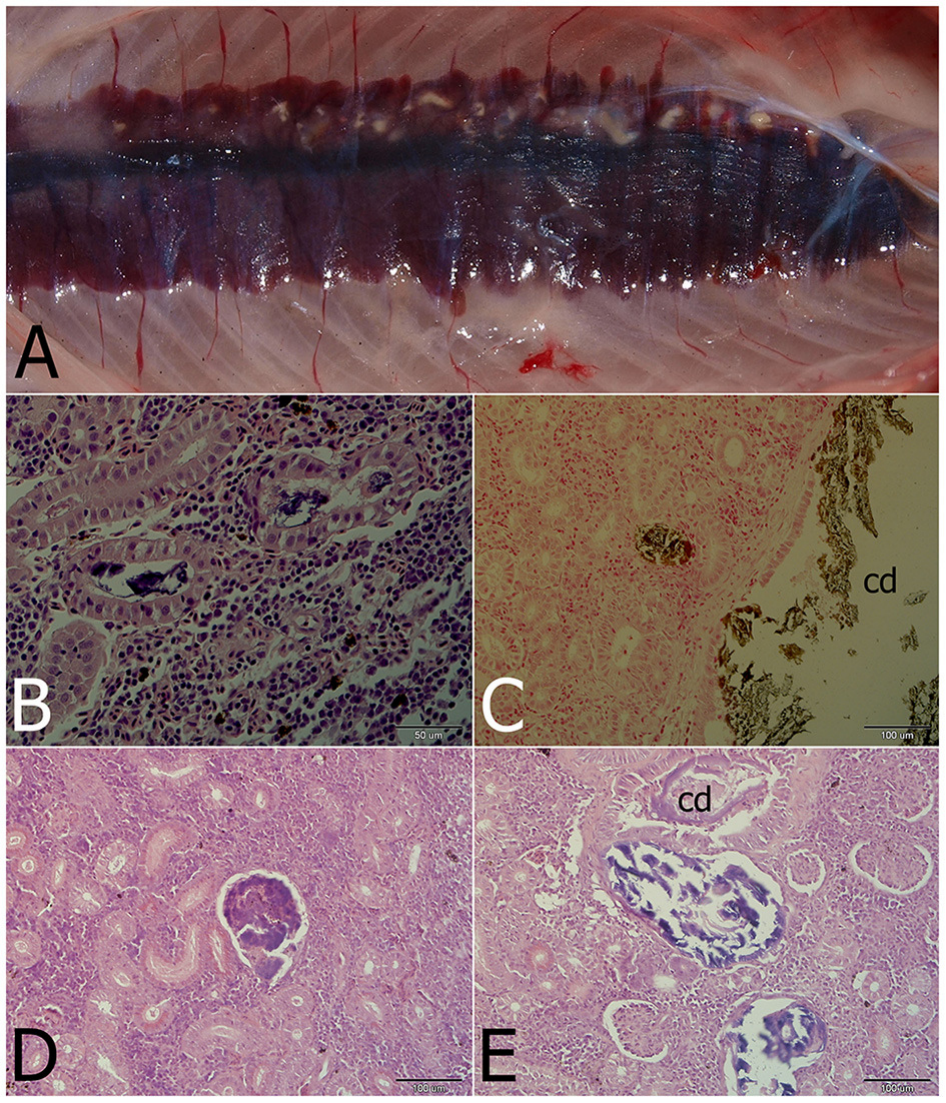
Nephrocalcinosis in a rainbow trout. **(A)** Macroscopically visible mineral deposits (white patches) in kidney parenchyma. **(B)** Renal tubules with strongly basophilic luminal deposits seen in the H&E-stained section. **(C)** Calcium deposits visualized by von Kossa staining in the convoluted tubule and the collecting duct (cd). **(D)** Advanced changes of kidney parenchyma associated with calcium deposits located probably in the former glomerulus. **(E)** Regressive changes of the collecting duct (cd) and/or renal tubules with mineralized luminal content differing in staining properties (H&E).

Gross examination revealed pronounced kidney lesions with marbling (n = 14), whitish mottling appearance and presence of renal casts (*n* = 9) (Figure 1A). Pathology findings also included skin lesions up to 0.5 cm diameter, with petechiae, and coelomic cavity distension (*n* = 7), while other fish showed swollen gills (*n* = 5) and swim bladder over-inflation (*n* = 4). Microscopically, renal tubules, and collecting and excretory ducts contained basophilic deposits (Figures 1B, E). Mild changes were characterized by presence of small mineral deposits in collecting ducts and tubules without damage to their epithelium (*n* = 7). Moderate changes were characterized by damage to the tubular wall (i.e., vacuolar degeneration) (*n* = 6). In more severely affected kidneys, necrosis of the tubular epithelium and regressive changes of collecting ducts were seen, including changes in glomeruli (i.e., dilatation of the glomerular space, thickening of the parietal layer and mineral deposits in glomeruli; Figures 1D, E; *n* = 6). Interstitial fibrosis was observed in the most severe cases of this study (*n* = 2). Mineral deposits were demonstrated by von Kossa staining (Figure 1C). The observed gross and microscopic pathological findings were consistent with nephrocalcinosis. No pathological findings were seen in healthy fish.

Univariate analysis revealed significant differences in 15 of 21 hematology and blood chemistry values measured in nephrocalcinosis-affected and healthy rainbow trout (Table 2). Hematology findings showed that, while red and white blood cell counts, hemoglobin and mean corpuscular hemoglobin concentrations had decreased in the nephrocalcinosis-affected group, mean corpuscular volume and mean corpuscular hemoglobin concentration had increased. Of the biochemical variables, Na, P and cholesterol levels had declined compared to the healthy group, while triglycerides had increased. Likewise, all acid-base balance variables had increased significantly (Table 2).

**Table 2:** Hematology and blood chemistry values measured in nephrocalcinosis (N)-affected and healthy rainbow trout.

**Variable**	**N-affected fish**	**Healthy fish**
	***N*** = **21**	***N*** = **8**
Hematocrit (L/L)	0.34 ± 0.06	0.36 ± 0.04
Hemoglobin (g/L)	73.09 ± 14.56^*^	87.33 ± 12.34
Red blood cell count (T/L)	0.79 ± 0.19^**^	1.16 ± 0.23
Mean corpuscular volume (fL)	450.57 ± 72.51^**^	314.18 ± 55.97
Mean corpuscular hemoglobin (pg)	95.23 ± 19.05^*^	76.78 ± 14.07
Mean corpuscular hemoglobin conc (g/L)	0.21 ± 0.02^**^	0.24 ± 0.03
White blood cell count (G/L)	17.33 ± 5.53^**^	30.00 ± 6.63
Na (mmol/L)	136.85± 1.80^**^	141.21 ± 1.53
K (mmol/L)	6.14 ± 0.77	6.48 ± 0.29
Cl^−^ (mmol/L)	112.23 ± 1.96	113.71 ± 1.71
Ca (mmol/L)	2.23 ± 0.30	2.42 ± 0.14
P (mmol/L)	3.74 ± 0.59^**^	4.53 ± 0.44
Glucose (mmol/L)	4.19 ± 1.08	4.07 ± 0.47
Triglycerides (mmol/L)	3.16 ± 1.35^**^	1.47 ± 0.59
Cholesterol (mmol/L)	5.62 ± 1.13^*^	6.79 ± 1.16
Total protein (g/L)	31.07 ± 4.60	30.12 ± 2.48
pH	7.32 ± 0.05^**^	6.97 ± 0.04
pCO_2_ (kPa)	9.31 ± 1.93^**^	2.14 ± 0.27
tCO_2_ (mmol/L)	38.65 ± 4.68^**^	4.90 ± 0.00
HCO_3_ (mmol/L)	36.55 ± 4.29^**^	3.69 ± 0.27
Base Excess (mmol/L)	10.53 ± 4.43^**^	−28.12 ± 0.83

Values represent mean ± standard deviation, ^*^p < 0.05, ^**^p < 0.01 when comparing N-affected fish against the healthy fish.

**Figure 2:**
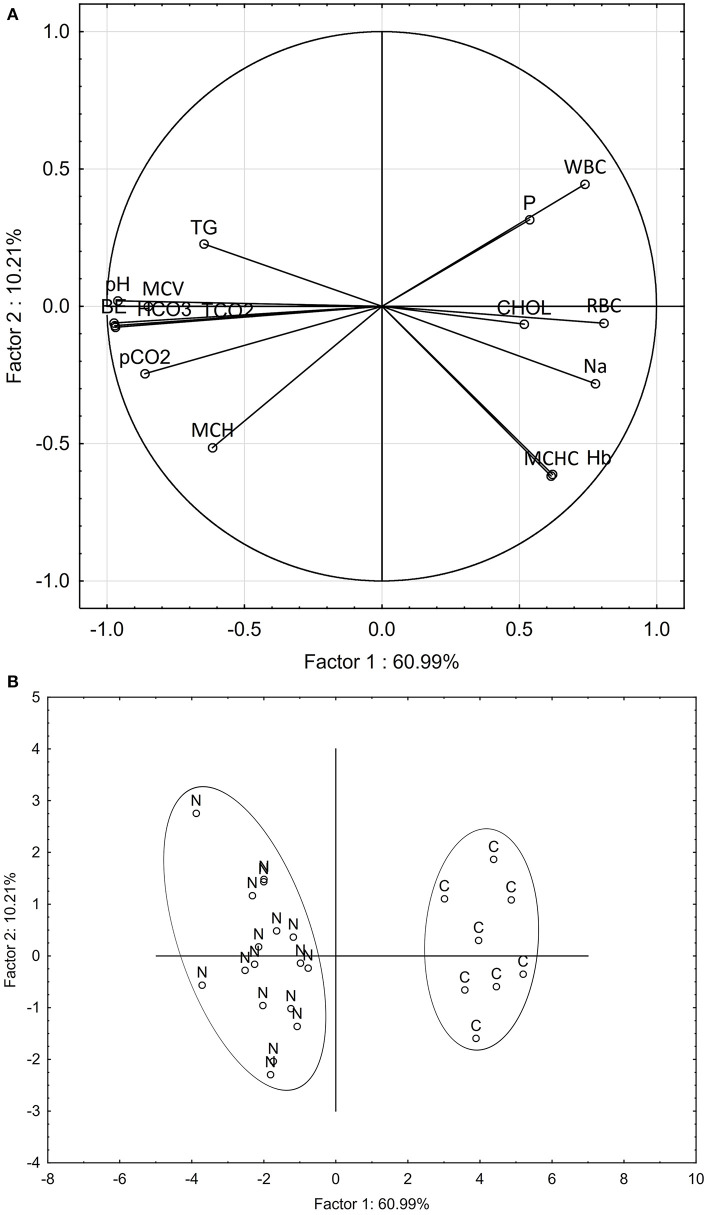
Principal component analysis discriminating between nephrocalcinosis (N)-affected and healthy (C) fish (*Oncorhynchus mykiss*), based on all blood profile parameters detected as altered using univariate analysis. Component weight **(A)** and component score **(B)** plots are based on multiple blood chemistry and hematology variables. BE, base excess; CHOL, cholesterol; Hb, hemoglobin; HCO_3_, bicarbonate; MCH, mean corpuscular hemoglobin; MCHC, mean corpuscular hemoglobin concentration; MCV, mean corpuscular volume; Na, sodium; P, phosphorus; pCO_2_, partial pressure of carbon dioxide; pH, potential of hydrogen; RBC, red blood cell count; WBC, white blood cell count; tCO_2_, total dissolved CO_2_; TG, triglycerides.

Multivariate analysis of patterns in blood chemistry and hematology variables clearly discriminated the nephrocalcinosis-affected trout from the healthy fish (Figure 2). Based on variable component weights, discrimination between the diseased and healthy fish was driven mostly by pH, tCO_2_, HCO_3_, and base excess (Figure 2A). The PCA analysis separated nephrocalcinosis-affected and healthy fish along the first and the second principal components axes, explaining 60.99 and 10.21% of variation, respectively (Figure 2B). Exclusion of correlated variables from the multivariate analysis to reduce the number of blood profile parameters for only those indicative of acid-base and electrolyte imbalance and white and red blood cell alteration increased the variation explained to 80.54 and 8.19%, respectively.

## 4. Discussion

Water quality plays a key role in the pathophysiology of fish diseases (2). In the present study, fish developing signs of nephrocalcinosis were found to have been exposed to increased levels of O_2_ and CO_2_, compared with a nearby control aquacultural facility. Increased levels of aquatic O_2_ in stocked tanks causes an increase in fish metabolic O_2_ consumption rate, with a subsequent rise in environmental CO_2_ due to tissue respiration (5, 8, 13). Hyperoxia results in hypoventilation of fish, causing retention of carbon dioxide with resultant respiratory acidosis (41). Indeed, levels of dissolved CO_2_ measured in the affected aquacultural facility had reached the upper limit considered safe for salmonid fish (6), causing the pH of the water to drop (42). Despite this, the pH remained within the optimal range (6.5–8.0) for freshwater fish species (9). Lower NO_3_, together with detectable levels of NH_4_ and NO_2_, the latter slightly over the maximum recommended concentration of 0.1 mg/L (43), suggest that the nitrifying bacteria used to convert excreted N waste products in the biological filters of the affected aquacultural facility were not functioning effectively (9). Fortunately, the Cl^−^ concentration was high enough to prevent uptake of NO_2_ through the gills, thereby preventing any signs of toxicity associated with methemoglobinemia (44).

While the typical arterial blood pH in teleost fishes ranges between 7.6 and 7.9, depending on the temperature (45, 46), the pH of intracellular fluid compartments varies between 7.0 and 7.4, with plasma pH being ~0.6 higher than intracellular values (47). It should be noted, that blood pH in this study was measured based on samples taken from punctured *caudalis* vessels (i.e., probably representing a mixture of arterial and venous blood) along with measurements taken from whole blood samples. Surprisingly, however, fish from both the affected and control facilities had blood pH levels lower than those typical for rainbow trout (45), suggesting acidosis. Furthermore, blood pH in fish from the control facility was even lower (6.97 ± 0.04) than that for the nephrocalcinosis-affected fish (7.32 ± 0.05). In this case, it is likely that other acid-base balance parameters came into play, suggesting metabolic compensation of blood pH in the nephrocalcinosis-affected fish.

Both univariate and multivariate analyses, used to reveal blood profile alterations in nephrocalcinosis-affected fish, indicated significant elevations in all acid-base balance variables, with 15 of the 21 hematology and blood chemistry parameters measured being significantly different from the healthy fish. As Chen et al. (21) have previously shown, however, a reduced number of blood profile parameters indicative of acid-base and electrolyte imbalance and white and red blood cell alteration should still be sufficient to discriminate and separate nephrocalcinosis-affected fish using multivariate analysis.

The acid-base parameters observed in our nephrocalcinosis-affected fish, i.e., lowered blood pH, together with elevated levels of CO_2_, HCO_3_, and base excess, are characteristic of respiratory acidosis with metabolic compensation (48). While fish gills are of critical importance for acid-base homeostasis, the kidneys play a significant role in metabolic compensation (49, 50). Hyponatremia is most probably associated with disrupted functioning of kidneys unable to adequately handle Na excretion (48). Reduced levels of Na have also been reported for CO_2_-exposed fish in an experimental study with Atlantic salmon (*Salmo salar* L.) smolts (13). Similar findings in Atlantic salmon were also observed by Klykken et al. (34). However, in contrast to Fivelstad et al. (12) and Klykken et al. (34), we did not register any significant drop in blood Cl^−^ concentrations.

The nephrocalcinosis-affected fish in this study also showed signs of hypophosphatemia and normocalcemia. Both the Ca and P metabolisms act in concert, with solubility for both being pH-dependent, meaning increased solubility in acidic environments and precipitation of calcium phosphate [Ca_3_(PO_4_)_2_] salts in alkaline conditions (49). As has previously been shown for rainbow trout, renal responses to acidosis involve phosphate excretion (51). In contrast, Klykken et al. (34) observed increased concentrations of Ca and P in blood plasma in 37% and 16% of fish affected by nephrocalcinosis (respectively), with only 8 and 3% with lower concentrations compared to the normal interval. Importantly, our data suggest that mineral deposition in the kidney may be a consequence of the increase in pH associated with metabolic compensation of acidosis.

Some of the blood profile parameters measured in this study, such as glucose, triglycerides, and cholesterol, reflect the nutrition, feeding activity, and/or energy mobilization of the fish being studied (52, 53). The low performance of fish exposed to hypercapnic conditions can be related to an acidosis-induced reduction in hemoglobin-O_2_ affinity (the Bohr effect) and O_2_-carrying capacity (the Root effect), and subsequent changes to feed intake and growth (11, 12, 14, 24, 54). The kidney interstitium is important for hematopoiesis in fish (55), thus nephrocalcinosis can affect blood cell counts and other hematology parameters as seen in the present study. Although there are some limitations to the use of blood analyses as a diagnostic tool (lack of standardized reference ranges), the comparison of healthy and nephrocalcinosis-affected fish can still be very informative to understand the mechanisms of the disease and may facilitate further monitoring.

The gross and microscopic findings were consistent with mild to moderate semi-quantitative scores, as described elsewhere (11, 12, 21, 25, 27, 34, 56). Even though severe cases of nephrocalcinosis may be of a highly suggestive appearance, kidney infections eliciting similar renal pathology (e.g., calcifying inflammatory lesions, granulomatous nephritis) must first be differentially ruled out (57–60).

To conclude, reaching the final diagnosis of nephrocalcinosis in the rainbow trout facility described here was not as straightforward as one would wish. When listing the issues making diagnosis difficult, it is first necessary to consider the creeping onset of the problem, which manifested itself as low performance and low-level mortality. Further important diagnostic steps included obtaining comparative data for water quality and fish blood profiles from a healthy control aquacultural facility, excluding infections with salmonid fish pathogens and/or heavy infestations with parasites, and careful examination of necropsy findings.

Major events in the pathophysiology of fish nephrocalcinosis in this case can be reconstructed as follows: aquatic environment hyperoxia and hypercapnia → blood hypercapnia → blood acid-base perturbation → respiratory acidosis → kidney P excretion → metabolic compensation of acid-base imbalance → blood HCO_3_ elevation → a rise in blood pH → calcium phosphate precipitation and deposition in tissues.

## Data availability statement

The original contributions presented in the study are included in the article/Supplementary material, further inquiries can be directed to the corresponding author.

## Ethics statement

The animal study was reviewed and approved by Ethical Committee of the University of Veterinary Sciences Brno, Czech Republic (REFNO: ES_11-2022_Necas). Written informed consent was obtained from the owners for the participation of their animals in this study.

## Author contributions

HM, MP, and JP conceived the study. JP and MP acquired funding. LP, IP, HM, IM, JM, VP, RK, and OM performed field and/or laboratory investigations. HM, JP, and MP analyzed the data and wrote the first draft of the manuscript. All authors contributed critical comments on manuscript drafts and gave final approval for its publication.

## References

[1] FAO. The State of World Fisheries and Aquaculture 2020. Sustainability in action. Rome: Food and Agriculture Organization (2020). 244 p.

[2] Roberts RJ. The Aquatic Environment. In:Roberts RJ, editor. Fish Pathology, 4th edition. London: Wiley-Blackwell (2012). p. 1–16. 10.1002/9781118222942.ch1

[3] Ellis T North B Scott AP Bromage NR Porter M Gadd D. The relationships between stocking density and welfare in farmed rainbow trout. J Fish Biol. (2002) 61:493–531. 10.1111/j.1095-8649.2002.tb00893.x

[4] Martins CIM Eding EH Verdegem MCJ Heinsbroek LTN Schneider O Blancheton JP. New developments in recirculating aquaculture systems in Europe: A perspective on environmental sustainability. Aquac Eng. (2010) 43:83–93. 10.1016/j.aquaeng.2010.09.002

[5] Skov PV. CO[[sb]]2[[/s]] in aquaculture. In:Grosell M Munday PL Farrell AP Brauner CJ, editors. Fish Physiology. 37. Academic Press (2019). p. 287–321.

[6] EFSA. Animal welfare aspects of husbandry systems for farmed Atlantic salmon—scientific opinion of the panel on animal health and welfare. EFSA J. (2008) 736:1–31. 10.2903/j.efsa.2008.736 PMC10193664 37213864

[7] Mota VC Nilsen TO Gerwins J Gallo M Kolarevic J Krasnov A. Molecular and physiological responses to long-term carbon dioxide exposure in Atlantic salmon (*Salmo salar*). Aquaculture. (2020) 519:734715. 10.1016/j.aquaculture.2019.734715

[8] MacIntyre CM Ellis T North BP Turnbull JF. The Influences of Water Quality on the Welfare of Farmed Rainbow Trout: A Review. In:Branson EJ, editor. Fish Welfare. UK: Wiley-Blackwell (2008). p. 150–84. 10.1002/9780470697610.ch10

[9] Pounder DB Smith SA. Water quality and environmental issues. In:Smith SA, editor. Fish diseases and medicine. Boca Raton, FL, USA: CRC Press (2019). p. 27–45. 10.1201/9780429195259-2

[10] Ellis RP Urbina MA Wilson RW. Lessons from two high CO[[sb]]2[[/s]] worlds – future oceans and intensive aquaculture. Glob Chang Biol. (2017) 23:2141–48. 10.1111/gcb.13515 27762490 PMC5434897

[11] Fivelstad S Olsen AB Åsgård T Baeverfjord G Rasmussen T Vindheim T. Long-term sublethal effects of carbon dioxide on Atlantic salmon smolts (*Salmo salar* L): ion regulation, haematology, element composition, nephrocalcinosis and growth parameters. Aquaculture. (2003) 215:301–19. 10.1016/S0044-8486(02)00048-0

[12] Fivelstad S Hosfeld CD Medhus RA Olsen AB. Kvamme K. Growth and nephrocalcinosis for Atlantic salmon (Salmo salar L) post-smolt exposed to elevated carbon dioxide partial pressures. Aquaculture. (2018) 482:83–9. 10.1016/j.aquaculture.2017.09.012

[13] Hosfeld CD Engevik A Mollan T Lunde TM Waagbø R Olsen AB. Long-term separate and combined effects of environmental hypercapnia and hyperoxia in Atlantic salmon (*Salmo salar* L) smolts. Aquaculture. (2008) 280:146–53. 10.1016/j.aquaculture.2008.05.009

[14] Smart GR Knox D Harrison JG Ralph JA Richard RH Cowey CB. Nephrocalcinosis in rainbow trout *Salmo gairdneri* Richardson; the effect of exposure to elevated CO[[sb]]2[[/s]] concentrations. J Fish Dis. (1979) 2:279–89. 10.1111/j.1365-2761.1979.tb00170.x

[15] Gelwicks KR Zafft DJ Bobbitt JP. Efficacy of carbonic acid as an anesthetic for rainbow trout. N Am J Fish Manag. (1998) 18:432.

[16] Neiffer DL Stamper MA. Fish sedation, analgesia, anesthesia, and euthanasia: considerations, methods, and types of drugs. ILAR J. (2009) 50:343–60. 10.1093/ilar.50.4.343 19949251

[17] Treanor HB Ray AM Layhee M Watten BJ Gross JA Gresswell RE. Using Carbon Dioxide in Fisheries and Aquatic Invasive Species Management. Fisheries. (2017) 42:621–28. 10.1080/03632415.2017.138390332813922

[18] Jutfelt F Bresolin de. Souza K, Vuylsteke A, Sturve J. Behavioural disturbances in a temperate fish exposed to sustained high-CO[[sb]]2[[/s]] levels. PLoS ONE. (2013) 8:e65825. 10.1371/journal.pone.0065825 23750274 PMC3672104

[19] Graff IE Waagbo R Fivelstad S Vermeer C Lie O Lundebye AK. multivariate study on the effects of dietary vitamin K, vitamin D[[sb]]3[[/s]] and calcium, and dissolved carbon dioxide on growth, bone minerals, vitamin status and health performance in smolting Atlantic salmon *Salmo salar* L. J Fish Dis. (2002) 25:599–614. 10.1046/j.1365-2761.2002.00403.x

[20] Gil Martens L Witten PE Fivelstad S Huysseune A Sævareid B Vikeså V. Impact of high water carbon dioxide levels on Atlantic salmon smolts (*Salmo salar* L): Effects on fish performance, vertebrae composition and structure. Aquaculture. (2006) 261:80–8. 10.1016/j.aquaculture.2006.06.031

[21] Chen C-Y Wooster GA Getchell RG Bowser PR Timmons MB. Nephrocalcinosis in nile tilapia from a recirculation aquaculture system: a case report. J Aquat Anim Health. (2001) 13:368–72. 10.1577/1548-8667(2001)013<0368:NINTFA>2.0.CO;2

[22] Chen C-Y Wooster GA Getchell RG Bowser PR Timmons MB. Blood chemistry of healthy, nephrocalcinosis-affected and ozone-treated tilapia in a recirculation system, with application of discriminant analysis. Aquaculture. (2003) 218:89–102. 10.1016/S0044-8486(02)00499-4

[23] Erkinharju T Dalmo RA Hansen M Seternes T. Cleaner fish in aquaculture: review on diseases and vaccination. Rev Aquac. (2021) 13:189–237. 10.1111/raq.12470

[24] Fivelstad S Haavik H Lovik G. Olsen AB. Sublethal effects and safe levels of carbon dioxide in seawater for Atlantic salmon postsmolts (*Salmo salar* L): ion regulation and growth. Aquaculture. (1998) 160:305–16. 10.1016/S0044-8486(97)00166-X

[25] Harrison JG Richards RH. The pathology and histopathology of nephrocalcinosis in rainbow trout *Salmo gairdneri* Richardson in fresh water. J Fish Dis. (1979) 2:1–12. 10.1111/j.1365-2761.1979.tb00134.x

[26] Klykken C Boissonnot L Reed AK Whatmore P Attramadal K Olsen RE. Gene expression patterns in Atlantic salmon (*Salmo salar*) with severe nephrocalcinosis. J Fish Dis. (2022) 45:1645–58. 10.1111/jfd.13687 35862221 PMC9796406

[27] McHugh KJ Van Dyk JC Weyl OLF Smit NJ. First report of nephrocalcinosis in a wild population of *Mugil cephalus* L. and Myxus capensis (Valenciennes). J Fish Dis. (2013) 36:887–9. 10.1111/jfd.12101 23496659

[28] Fornshell G. Rainbow trout—challenges and solutions. Rev Fish Sci. (2002) 10:545–57. 10.1080/20026491051785

[29] Burgos-Aceves MA Lionetti L Faggio C. Multidisciplinary haematology as prognostic device in environmental and xenobiotic stress-induced response in fish. Sci Total Environ. (2019) 670:1170–83. 10.1016/j.scitotenv.2019.03.275 31018433

[30] Fazio F. Fish hematology analysis as an important tool of aquaculture: a review. Aquaculture. (2019) 500:237–42. 10.1016/j.aquaculture.2018.10.030

[31] Rozas-Serri M Correa R Walker-Vergara R Coñuecar D Barrientos S Leiva C. Reference intervals for blood biomarkers in farmed atlantic salmon, coho salmon and rainbow trout in chile: promoting a preventive approach in aquamedicine. Biology. (2022) 11:1066. 10.3390/biology11071066 36101444 PMC9312075

[32] Pikula J Pojezdal L Papezikova I Minarova H Mikulikova I Bandouchova H. Carp edema virus infection is associated with severe metabolic disturbance in fish. Front Vet Sci. (2021) 8:679970. 10.3389/fvets.2021.679970 34095283 PMC8169968

[33] Svobodova Z Vykusova B Modra H Jarkovsky J Smutna M. Haematological and biochemical profile of harvest-size carp during harvest and post-harvest storage. Aquac Res. (2006) 37:959–65. 10.1111/j.1365-2109.2006.01511.x

[34] Klykken C Reed AK Dalum AS Olsen RE Moe MK Attramadal KJK. Physiological changes observed in farmed Atlantic salmon (*Salmo salar* L.) with nephrocalcinosis. Aquaculture. (2022) 554:738104. 10.1016/j.aquaculture.2022.738104

[35] Pojezdal L Adamek M Syrova E Steinhagen D Minarova H Papezikova I. Health Surveillance of Wild Brown Trout (*Salmo trutta fario*) in the Czech Republic Revealed a Coexistence of Proliferative Kidney Disease and Piscine *Orthoreovirus-3* Infection. Pathogens. (2020) 9:604. 10.3390/pathogens9080604 32722219 PMC7460431

[36] Palikova M Navrátil S CíŽek A Soukupová Z Lang Š Kopp R. Seasonal occurrence of diseases in a recirculation system for salmonid fish in the Czech Republic. Acta Vet Brno. (2014) 83:201–07. 10.2754/avb201483030201

[37] Palikova M Pojezdal L Davidova-Gerzova L Novakova V Pikula J Papezikova I. Carp edema virus infection associated gill pathobiome: a case report. J Fish Dis. (2022) 45:1409–17. 10.1111/jfd.13670 35708022

[38] Seidlova V Syrova E Minarova H Zukal J Balaz V Nemcova M. Comparison of diagnostic methods for *Tetracapsuloides bryosalmonae* detection in salmonid fish. J Fish Dis. (2021) 44:1147–53. 10.1111/jfd.13375 33837562 PMC8360006

[39] Zalud Z Kren J Spurny P Trnka M. Biological and technological aspects of sustainability of controlled ecosystems and their adaptability to climate change—indicators of ecosystem services. Folia Univ Agric et Silvic Mendel Brun. (2008) 4:75–116.

[40] APHA. Standard Methods for the Examination of Water and Wastewater, 20th edition. Washington DC: American Public Health Association, American Water Works Association, Water Environmental Federation (1998).

[41] McArley TJ Sandblom E Herbert NA. Fish and hyperoxia—From cardiorespiratory and biochemical adjustments to aquaculture and ecophysiology implications. Fish Fish. (2021) 22:324–55. 10.1111/faf.12522

[42] Randall DJ Wright PA. The interaction between carbon dioxide and ammonia excretion and water pH in fish. Can J Zool. (1989) 67:2936–42. 10.1139/z89-416

[43] Wedemeyer GA. Physiology of Fish in Intensive Culture Systems. New York: Springer New York (1996). 232 p. 10.1007/978-1-4615-6011-1

[44] Lewis WM Morris DP. Toxicity of nitrite to fish: a review. Trans Am Fish Soc. (1986) 115:183–95.

[45] Burton RF. The dependence of normal arterial blood pH on sodium concentration in teleost fish. Comp Biochem Physiol Part A Physiol. (1996) 114:111–16. 10.1016/0300-9629(95)02102-7 7915647

[46] Randall DJ. “Transport and exchange of respiratory gases in the blood | Carbon Dioxide Transport and Excretion,” In:Farrell AP, ed. Encyclopedia of Fish Physiology. San Diego: Academic Press (2011). p. 909–15. 10.1016/B978-0-12-374553-8.00027-7

[47] Claiborne JB Edwards SL Morrison-Shetlar AI. Acid-base regulation in fishes: cellular and molecular mechanisms. J Exp Zool. (2002) 293:302–19. 10.1002/jez.10125 12115903

[48] Campbell TW. “Clinical Chemistry of Fish and Amphibians,” In:Thrall MA Weiser G Allison RW Campbell TW, eds. Veterinary Hematology and Clinical Chemistry, 2nd Edition. Hoboken, New Jersey, USA: Wiley-Blackwell (2012). p. 607–14.

[49] Perry SF Gilmour KM. Acid-base balance and CO[[sb]]2[[/s]] excretion in fish: unanswered questions and emerging models. Respir Physiol Neurobiol. (2006) 154:199–215. 10.1016/j.resp.2006.04.010 16777496

[50] Takvam M Wood CM Kryvi H Nilsen TO. Ion transporters and osmoregulation in the kidney of teleost fishes as a function of salinity. Front Physiol. (2021) 12:664588. 10.3389/fphys.2021.664588 33967835 PMC8098666

[51] Wood CM Milligan CL Walsh PJ. Renal responses of trout to chronic respiratory and metabolic acidoses and metabolic alkalosis. Am J Physiol. (1999) 277:R482–92. 10.1152/ajpregu.1999.277.2.R482 10444555

[52] Congleton JL Wagner T. Blood-chemistry indicators of nutritional status in juvenile salmonids. J Fish Biol. (2006) 69:473–90. 10.1111/j.1095-8649.2006.01114.x

[53] Pottinger TG Rand-Weaver M Sumpter JP. Overwinter fasting and re-feeding in rainbow trout: plasma growth hormone and cortisol levels in relation to energy mobilisation. Comp Biochem Physiol B Biochem Mol Biol. (2003) 136:403–17. 10.1016/S1096-4959(03)00212-4 14602149

[54] Berenbrink M. “Transport and exchange of respiratory gases in the blood. Root Effect: Molecular Basis, Evolution of the Root Effect and Rete Systems,” In Farrell AP, ed. Encyclopedia of Fish Physiology. San Diego: Academic Press (2011). p. 935–43. 10.1016/B978-0-12-374553-8.00117-9

[55] Kobayashi I Katakura F Moritomo T. Isolation and characterization of hematopoietic stem cells in teleost fish. Dev Comp Immunol. (2016) 58:86–94. 10.1016/j.dci.2016.01.003 26801099

[56] Klykken C Dalum AS Reed AK Attramadal K Olsen RE Boissonnot L. Radiological detection of nephrocalcinosis in farmed Atlantic salmon *Salmo salar* L. J Fish Dis. (2022) 45:1883–8. 10.1111/jfd.13704 35964249 PMC9804365

[57] Beraldo P Berton D Giavenni R Galeotti M. First report on proliferative kidney disease (PKD) in marble trout (*Salmo trutta marmoratus*, Cuvier 1817). Bull Eur Ass Fish Pathol. (2006) 26:143–50.

[58] Bettge K Wahli T Segner H Schmidt-Posthaus H. Proliferative kidney disease in rainbow trout: time- and temperature-related renal pathology and parasite distribution. Dis Aquat Organ. (2009) 83:67–76. 10.3354/dao01989 19301638

[59] Ferguson HW Needham EA. Proliferative kidney disease in rainbow trout *Salmo gairdneri* Richardson. J Fish Dis. (1978) 1:91–108. 10.1111/j.1365-2761.1978.tb00008.x6474776

[60] Palikova M Papezikova I Markova Z Navratil S Mares J Mares L. Proliferative kidney disease in rainbow trout (*Oncorhynchus mykiss*) under intensive breeding conditions: Pathogenesis and haematological and immune parameters. Vet Parasitol. (2017) 238:5–16. 10.1016/j.vetpar.2017.03.003 28291603

