# Supplementary material for: Nephrocalcinosis in farmed salmonids: diagnostic challenges associated with low performance and sporadic mortality
Source: Front Vet Sci. 2023 Apr 20;10:1121296. doi: 10.3389/fvets.2023.1121296 (PMC10157097; doi:10.3389/fvets.2023.1121296)
Supplement: Supplementary file 1 [file Data_Sheet_1.PDF]

Supplementary Material

Nephrocalcinosis in farmed salmonids: diagnostic challenges associated with low performance and sporadic mortality

Hana Minarova, Miroslava Palikova, Radovan Kopp, Ondrej Maly, Jan Mares, Ivana Mikulikova, Ivana Papezikova, Vladimir Piacek, Lubomir Pojezdal, Jiri Pikula

*** Correspondence: Jiri Pikula** Corresponding Author: pikulaj@vfu.cz

Supplementary Data

Hematology and blood chemistry values measured in nephrocalcinosis-affected and healthy rainbow trout.

fish group	Na (mmol/L)	K (mmol/L)	Cl (mmol/L)	tCO_2 (mmol/L)	glucose (mmol/L)	haematocrit (L/L)	pH	pCO_2 (kPa)
Nephrocalcinosis 1	136,9	6,67	112,3			0,400		
Nephrocalcinosis 2	136,8	6,39	113,1	38,0	4,3	0,345	7,329	9,02
Nephrocalcinosis 3	135,9	4,56	111,4	32,0	4,0	0,370	7,294	8,36
Nephrocalcinosis 4	134,7	6,47	110,4	37,0	4,3	0,275	7,335	8,74
Nephrocalcinosis 5	136,8	6,87	112,1	35,0	5,1	0,355	7,277	9,48
Nephrocalcinosis 6	137,2	4,38	111,8	36,0	2,1	0,310	7,332	8,57
Nephrocalcinosis 7	136,6	5,81	113,3	31,0	2,3	0,355	7,329	7,54
Nephrocalcinosis 8	135,0	5,74	112,0	34,0	5,8	0,365	7,293	8,77
Nephrocalcinosis 9	139,5	6,01	115,7	35,0	4,4	0,320	7,330	8,49
Nephrocalcinosis 10	135,6	5,93	110,5	40,0	4,1	0,350	7,316	9,88
Nephrocalcinosis 11	135,6	5,93	110,5			0,400		
Nephrocalcinosis 12	134,1	6,56	108,6	41,0	2,5	0,130	7,470	4,16
Nephrocalcinosis 13	138,7	6,72	112,3			0,380		
Nephrocalcinosis 14	136,8	7,01	112,8	41,0	4,8	0,350	7,322	10,00
Nephrocalcinosis 15	140,2	6,95	115,4	38,0	4,3	0,340	7,312	9,53
Nephrocalcinosis 16	137,0	6,53	112,8	48,0	5,1	0,340	7,285	12,56
Nephrocalcinosis 17	138,1	6,56	113,6	39,0	3,4	0,325	7,336	9,10

Nephrocalcinosis 18	140,9	4,65	115,2	43,0	4,7	0,420	7,238	12,52
Nephrocalcinosis 19	135,3	6,13	111,4	45,0	5,6	0,420	7,307	11,45
Nephrocalcinosis 20	137,8	6,82	113,4			0,330		
Nephrocalcinosis 21	135,3	6,23	108,2	44,0	4,5	0,370	7,347	10,09
Control 1	140,1	6,19	112,1	4,9	4,3	0,330	6,951	2,12
Control 2	142,2	6,36	113,8	4,9	3,1	0,360	6,993	2,06
Control 3	143,5	6,95	115,8	4,9	4,4	0,440	6,916	2,21
Control 4	142,4	6,53	113,9	4,9	4,1	0,330	7,034	1,78
Control 5	141,1	6,56	112,8	4,9	3,8	0,350	7,025	1,99
Control 6	138,5	6,34	113,8	4,9	4,7	0,370	6,924	2,73
Control 7	140,9	6,13	114,7	4,9	4,1	0,320	6,956	2,06
Control 8	141,0	6,82	112,8	4,9	4,1	0,350	6,968	2,21

HCO_3 (mmol/L)	base excess (mmol/L)	haemoglobin (g/L)	red blood cell count (T/L)	total protein (g/L)	P (mmol/L)	triglycerides (mmol/L)	Ca (mmol/L)
		97,31	0,62	36,84	3,41	3,50	2,60
35,6	10	85,33	0,86	37,06	4,28	4,32	2,34
30,4	4	79,52	0,93	34,80	3,81	2,79	2,31
35,0	9	65,72	0,62	32,42	3,09	4,82	2,07
33,2	6	74,80	0,87	32,25	4,17	4,01	2,34
34,1	8	77,34	0,71	24,50	3,12	1,11	1,93
29,8	4	57,01	0,70	28,21	3,48	1,55	2,57
31,8	5	80,61	0,94	31,21	4,05	1,79	2,53
33,6	8	76,25	0,76	23,43	3,65	1,28	2,36
37,8	12	68,08	0,72	32,51	4,42	5,04	2,29
		77,88	1,04	32,51	4,42	5,04	2,29
39,1	15	23,24	0,23	28,49	4,08	3,34	2,14
		75,52	1,03	38,66	2,33	0,63	1,44
38,9	13	64,81	0,96	30,49	3,79	4,25	2,15
36,2	10	70,99	0,66	30,69	3,34	2,46	1,95
44,8	18	70,62	0,61	28,21	4,66	4,17	2,46
36,5	11	65,18	0,75	28,02	3,62	3,39	1,94

40,1	13	88,60	0,91	34,76	3,32	2,89	2,63
42,9	17	82,79	1,06	35,76	4,25	4,29	2,62
		73,53	0,76	20,86	2,96	2,01	1,81
41,5	16	79,70	0,83	30,79	4,25	3,60	2,13
3,5	-29	70,08	0,84	26,26	4,72	0,89	2,44
3,7	-28	90,05	0,99	26,43	5,19	0,67	2,24
3,4	-29	105,30	1,23	32,94	4,79	1,06	2,63
3,6	-27	92,95	0,99	31,68	4,05	1,81	2,30
3,9	-27	84,97	1,39	31,54		1,70	
4,2	-28	100,58	1,51	29,98	4,14	1,58	2,50
3,4	-29	80,97	1,06	30,68	4,29	1,54	2,39
3,8	-28	73,71	1,31	31,45		2,53	

Cholesterol (mmol/L)	White blood cell count (G/L)	MCH (pg)	MCV (fL)	MCHC (g/L)
5,95	25	156,95	645,16	0,24
6,54	17	99,22	401,16	0,25
5,75	17	85,50	397,85	0,21
5,69	25	106,00	443,55	0,24
6,25	20	85,98	408,05	0,21
5,63	10	108,93	436,62	0,25
5,37	19	81,44	507,14	0,16
6,02	18	85,75	388,30	0,22
5,40	18	100,33	421,05	0,24
6,27	25	94,56	486,11	0,19
6,27	7	74,89	384,62	0,19
5,82	24	101,04	565,22	0,18
1,94	7	73,33	368,93	0,20
4,23	20	67,51	364,58	0,19
6,41	20	107,55	515,15	0,21
4,68	12	115,78	557,38	0,21
5,53	15	86,90	433,33	0,20

7,19	10	97,36	461,54	0,21
7,05	20	78,10	396,23	0,20
4,33	16	96,75	434,21	0,22
5,65	19	96,02	445,78	0,22
6,51	27	83,43	392,86	0,21
5,55	20	90,96	363,64	0,25
8,32	39	85,61	357,72	0,24
7,88	26	93,89	333,33	0,28
6,83	39	61,13	251,80	0,24
5,22	26	66,61	245,03	0,27
6,09	32	76,39	301,89	0,25
7,94	31	56,27	267,18	0,21
